# Tripartite genome of all species

**DOI:** 10.12688/f1000research.8008.1

**Published:** 2016-02-19

**Authors:** MengPing Long, TaoBo Hu

**Affiliations:** 1 Division of Life Science, Hong Kong University of Science and Technology, Hong Kong SAR, China; 2 Department of Neurology, Xiangya Hospital, Central South University, Changsha, China; 3 Applied Genomics Center, Hong Kong University of Science and Technology, Hong Kong SAR, China; 4 Department of Oncology, The Second Xiangya Hospital, Central South University, Changsha, China

**Keywords:** Increasing Functional Variation hypothesis, Maximum Genetic Diversity theory, Neutral theory, Evolution, Genome architecture, Gene regulation

## Abstract

Neutral theory has dominated the molecular evolution field for more than half a century, but it has been severely challenged by the recently emerged Maximum Genetic Diversity (MGD) theory. However, based on our recent work of tripartite human genome architecture, we found that MGD theory may have overlooked the regulatory but variable genomic regions that increase with species complexity. Here we propose a new molecular evolution theory named Increasing Functional Variation (IFV) hypothesis. According to the IFV hypothesis, the genome of all species is divided into three regions that are ‘functional and invariable’, ‘functional and variable’ and ‘non-functional and variable’. While the ‘non-functional and variable’ region decreases as species become more complex, the other two regions increase.

## Introduction

**Figure 1.**
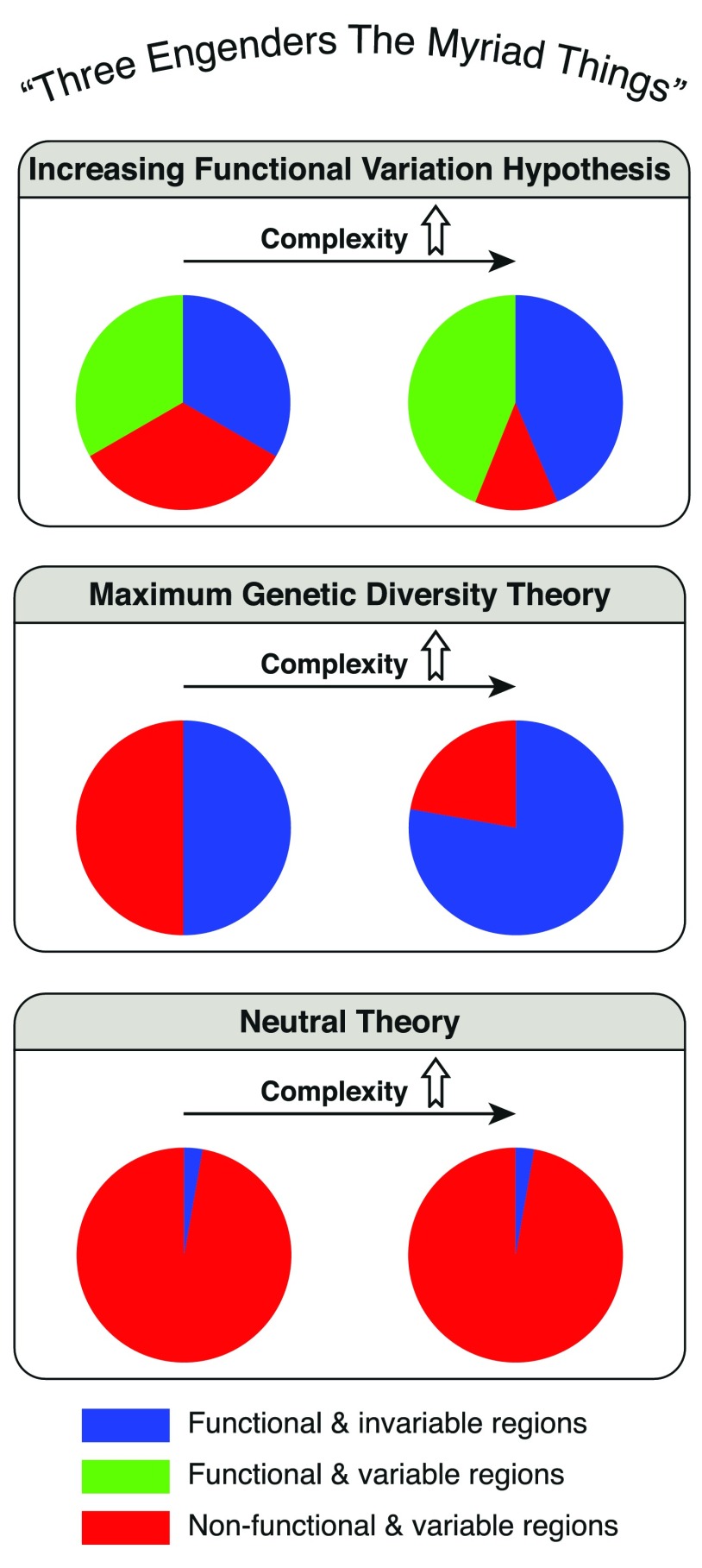
Comparison of IFV, MGD, and neutral theory. While the neutral theory and MGD theory analyze genome structure as bipartite, the IFV hypothesis adds an additional region which is the variable and functional gene regulatory region. As species complexity increases, the variable region of the genome would stay as variable according to neutral theory. While in MGD theory, as species complexity increases there would be less variable region. Unlike MGD theory, IFV hypothesis states that the functional variable region which contains gene regulatory elements would also increase with species complexity.

The structure and function of the genome have been a major question that all researchers want to solve. The current popular view of the genomic structure is represented by the neutral theory. The neutral theory states that the majority of the genome is variable and neutral ^1^. The variable property of these genomic regions would not change as the complexity of species increases ( Figure 1).

While in recent years, another theory called Maximum Genetic Diversity (MGD) provided unprecedented insights into the genome structure ^2– 5^. The MGD theory originated from blasting some conserved proteins such as cytochrome C and hemoglobin of different species. By computing the changeable sites of each species ^3^, Huang found that more complex species have less changeable sites in certain regions of the genome. Thus, MGD theory states that as the complexity of species increases, the genome would have more invariable regions and less variable regions ( Figure 1).

## IFV hypothesis

Here we proposed the Increasing Functional Variation (IFV) hypothesis inspired by both the MGD theory ^2^ and our recent work on human genome architecture ^6^. Recently, based on co-localization of various genomic features we divided the human genome into three parts, referred to as gene enriched (Genic) zones, gene regulatory elements enriched (Proximal) zones and non-functional features enriched (Distal) zones ^6^. We regard the Genic zones as mainly functional and invariable, and the Distal zones as mainly non-functional and variable. The Proximal zones that compose 31% of human genome contain the majority of gene regulatory elements including transcriptional factor binding sites (TFBSs) and are at the same time enriched with conserved indels. These features make Proximal zones functional and variable. It has been proven that as the complexity of species increase, there would be more gene regulatory region in the genome. Based on these two points, we propose that as the complexity of species increases, this variable part of the genome which contains functional regulatory elements would also increase. We call it the Increasing Functional Variation (IFV) hypothesis. Besides the variable gene regulatory region, the other part of the genome can be divided into two parts, the functional and invariable region and the non-functional and variable region. The alteration of these two parts with species complexity can be explained by MGD theory ( Figure 1). What the MGD theory lacks and IVF hypothesis complements is the existence of the variable and functional gene regulatory region in the genome. And according to the IFV hypothesis, as species complexity increases, the variable part of the genome would not simply decrease as stated by MGD theory. The differences between IVF hypothesis and MGD theory have been illustrated in Table 1.

**Table 1.** Comparison of IFV hypothesis and MGD theory.

	IFV	MGD
**Genome architecture**	Tripartite	Bipartite
**Types of variable** **region**	Two	One
**Alteration of variable** **region as species** **complexity increases**	Functional variable region increases while non-functional variable region decreases	Decreases

## Conclusions

MGD theory has refuted the idea stated by the neutral theory that the majority of the genome is neutral and variable among all species. Instead, it proved that the variable region of the genome would decrease as species become more complex.

However, MGD theory has its own limitation as pointed out by Ho shortly after the publication of MGD. As Ho has mentioned in her book ^7^, more complex species have more sequence diversity, which is needed for precise regulation of local somatic expression. Ho also stated that although MGD theory solved the paradoxes in molecular evolution, the diversity of complex species at somatic level can’t be explained by it. Our recent study ^6^ on human genome architecture discovered not only variable but also functional regions of the human genome. In an attempt to provide a more comprehensive view of genome structure and molecular evolution, we developed the IFV hypothesis based on our discovery of the variable property of the gene regulatory region.

Why would we develop this tripartite model of genome architecture across all species? As the ancient Chinese philosopher *Lao Tzu* stated in *Tao Te Ching* thousands of years ago that “ *three engenders the myriad things*”, which means “three” is the root of all things. If the truth of the universe is universal, we believe that the consistency between our tripartite genome architecture of all species and *Lao Tzu*’s philosophical thinking is not a coincidence.
