# Supplementary material for: Tripartite genome of all species
Source: F1000Res. 2016 Feb 19;5:195. [Version 1] doi: 10.12688/f1000research.8008.1 (PMC4911623; doi:10.12688/f1000research.8008.1)
Supplement: Peer review history

# Peer review: Referee response for version 1

**DOI:** 10.5256/f1000research.8617.r14116

**Published:** 2016-06-16

**Authors:** Alfredo Pulvirenti

**Affiliations:** 1 Department of Clinical and Experimental Medicine, University of Catania, Catania, Italy

The paper, in its current version, presents several major limitations. 

 The authors should provide more details about the theories.

 Furthermore, the rationale behind their IFV hypothesis should be given in a deeper way. Examples supporting the conclusions are strongly needed. The authors should also highlight the limitations and the opportunities of this hypothesis.

I have read this submission. I believe that I have an appropriate level of expertise to confirm that it is of an acceptable scientific standard, however I have significant reservations, as outlined above.

# Peer review: Referee response for version 1

**DOI:** 10.5256/f1000research.8617.r13461

**Published:** 2016-05-03

**Authors:** Rahul Banerjee

**Affiliations:** 1 Crystallography and Molecular Biology Division, Saha Institute of Nuclear Physics, Kolkata, India

The manuscript by Long and Hu is of considerable interest in the field of evolution of genomes. However the authors could consider the following points to improve the quality of the manuscript :

1. Is there any numerical measure of species complexity ?
2. On page 2, column 2, line 6
  'Huang found that more complex species have less changeable sites in certain regions of the genomes.'
  It would be nice if some more details could be included with regard to these sites.
3. The authors should be more elaborate as to why they considered the Genic zones to be mainly functional and invariable and the Distal zones as mainly non-functional and variable, and the Proximal zones functional and variable.
4. The note about Lao Tzu was also quite attractive and added some philosophical charm to the scientific note.

I have read this submission. I believe that I have an appropriate level of expertise to confirm that it is of an acceptable scientific standard.

# Peer review: Referee response for version 1

**DOI:** 10.5256/f1000research.8617.r12943

**Published:** 2016-03-29

**Authors:** Shaillay Dogra

**Affiliations:** 1 Vishuo BioMedical Pte Ltd, Singapore, Singapore

It would be nicer for the readers if there was more explanation and discussion in the text on the various different theories, what they imply, their pros and cons etc. Currently, it requires some background reading on these topics for the not so well-informed reader.

A detailed discussion of the basis of the IVF hypothesis, the basis of the proposals, its criticism etc. is suggested for a greater understanding of the author's ideas.

I have read this submission. I believe that I have an appropriate level of expertise to confirm that it is of an acceptable scientific standard, however I have significant reservations, as outlined above.
